# Molecular Characterization Reveals the Involvement of Calcium Dependent Protein Kinases in Abiotic Stress Signaling and Development in Chickpea (*Cicer arietinum*)

**DOI:** 10.3389/fpls.2022.831265

**Published:** 2022-04-12

**Authors:** Deepika Deepika, Nikita Poddar, Shailesh Kumar, Amarjeet Singh

**Affiliations:** ^1^Stress Signaling Lab, National Institute of Plant Genome Research, New Delhi, India; ^2^Bioinformatics Lab, National Institute of Plant Genome Research, New Delhi, India

**Keywords:** abiotic stress, CDPK, chickpea, gene expression, signaling, development

## Abstract

Calcium-dependent protein kinases (CDPKs) are a major group of calcium (Ca^2+)^ sensors in plants. CDPKs play a dual function of “Ca^2+^ sensor and responder.” These sensors decode the “Ca^2+^ signatures” generated in response to adverse growth conditions such as drought, salinity, and cold and developmental processes. However, knowledge of the CDPK family in the legume crop chickpea is missing. Here, we have identified a total of 22 *CDPK* genes in the chickpea genome. The phylogenetic analysis of the chickpea CDPK family with other plants revealed their evolutionary conservation. Protein homology modeling described the three-dimensional structure of chickpea CDPKs. Defined arrangements of α-helix, β-strands, and transmembrane-helix represent important structures like kinase domain, inhibitory junction domain, N and C-lobes of EF-hand motifs. Subcellular localization analysis revealed that CaCDPK proteins are localized mainly at the cytoplasm and in the nucleus. Most of the CaCDPK promoters had abiotic stress and development-related *cis*-regulatory elements, suggesting the functional role of CaCDPKs in abiotic stress and development-related signaling. RNA sequencing (RNA-seq) expression analysis indicated the role of the CaCDPK family in various developmental stages, including vegetative, reproductive development, senescence stages, and during seed stages of early embryogenesis, late embryogenesis, mid and late seed maturity. The real-time quantitative PCR (qRT-PCR) analysis revealed that several *CaCDPK* genes are specifically as well as commonly induced by drought, salt, and Abscisic acid (ABA). Overall, these findings indicate that the CDPK family is probably involved in abiotic stress responses and development in chickpeas. This study provides crucial information on the CDPK family that will be utilized in generating abiotic stress-tolerant and high-yielding chickpea varieties.

## Introduction

Chickpea, the world's second most important food legume, is majorly produced by South Asia. Importantly, as the largest producer of chickpea, India contributes about 70% (5.9 million tons (mt) annually) to the world's chickpeas production (Varshney et al., 2009). Chickpea seeds are of high nutrition value as they contain 20–30% crude protein, 40% carbohydrate, 3–6% oil, and an abundance of minerals, such as calcium, magnesium, potassium, phosphorus, iron, and zinc (Gil et al., 1996; Ibrikci et al., 2003). Unfortunately, due to continuously fluctuating and harsh environmental conditions in the semi-arid tropics where chickpea is majorly cultivated, its productivity is severely affected. An estimated 40–60% of annual global chickpea yield losses are accounted for by abiotic stress factors only. While drought inflicts severe damage and accounts for almost 50% of chickpea yield loss, temperature changes and soil salinity together account for about 25% of chickpea yield loss (Varshney et al., 2014). Consequently, a wide gap is developed between chickpea demand and supply. Importantly, damage to chickpea yield dents several chickpea-producing countries economically. Drought, cold, and salinity, respectively, are reported to cost ~1.3 billion, 186, and 354 million US dollars (Ryan, 1997). Abiotic stresses negatively impact the flower set, pollen viability, pod set/abortion, and retention. As these developmental stages determine the seed number in chickpea, a negative impact on them significantly hampers chickpea yield. Thus, identification of crucial stress-related genes and their utilization in breeding programs to generate stress-tolerant chickpea varieties are urgently required.

Abiotic stresses are known to elicit a profound increase in cytosolic calcium (Ca^2+)^ levels. The spatio-temporal accumulation of Ca^2+^ develops a specific “Ca^2+^ signature” in the form of Ca^2+^ spikes, waves, and oscillations. The Ca^2+^ signature encoded by a specific stimulus is decoded by various Ca^2+^ sensors, toward a specific response (Tang et al., 2020). Major Ca^2+^ sensors that have been identified and characterized in plants include calmodulin (CaM) and CaM-like proteins (CMLs) (Zeng et al., 2015), calcineurin B-like proteins (CBLs) (Luan, 2009), and Ca^2+^-dependent protein kinases (CDPKs) (Singh et al., 2017). Among these, CDPKs are one of the largest Ca^2+^ sensor groups in the plant kingdom and constitute a multi-gene family (Schulz et al., 2013; Xiao et al., 2017). A total of 34 CDPK have been found in the *Arabidopsis thaliana* genome (Cheng et al., 2002), 31 genes in the rice (*Oryza sativa*) genome (Ray et al., 2007), and 20 genes in the wheat (*Triticum aestivum* L.) genome (Li et al., 2008). CDPKs are unique enzymes with a dual function of a Ca^2+^ sensor and responder, attributed to their peculiar structural features. Differing from other Ca^2+^ sensors, CDPKs can sense, respond to, and translate Ca^2+^ signals into protein phosphorylation events (Poovaiah et al., 2013). Plant CDPKs are comprised of a conserved domain structure and are monomeric in nature. A variable N-terminal domain is followed by a ser/thr kinase domain and a CDPK activation domain (CAD). An auto-inhibitory region called as “inhibitory junction domain” and a calcium-binding CaM-like domain (CaM-LD) is located within CAD (Schulz et al., 2013). CaM-LD contains four elongation factors (EF) hand motifs that are responsible for Ca^2+^ binding. These EF hands are organized as N-terminal and C-terminal lobes (each consists of two EF hands) (Boudsocq and Sheen, 2013; Liese and Romeis, 2013). At the basal state, the C-terminal lobe shows high affinity to Ca^2+^ thus, it remains loaded with Ca^2+^ even at low Ca^2+^ concentration. The C-terminal lobe *via* interaction with auto-inhibitory junction maintains the kinase in an inactive state. The binding of Ca^2+^ induces a conformational change in the N-terminal lobe, which disrupts auto-inhibitory junction-kinase interaction. That removes auto-inhibition and leads to the activation of kinase (Boudsocq and Sheen, 2013). In plants, different CDPK isoforms have been found to exhibit distinct expression patterns, which possibly accounts for their functional specificity (Yang et al., 2017; Zhang et al., 2017). CDPK proteins localize in the cytosol and subcellular organelles, including the nucleus, plasma membrane, endoplasmic reticulum, tonoplast, mitochondria, and chloroplasts (Simeunovic et al., 2016). This suggests that CDPKs might target variable substrates throughout the plant cell. In plants, CDPKs have been involved in regulating important functions, including biotic and abiotic responses, hormone signaling, and development (Schulz et al., 2013). Knowledge of plants' CDPK functions has been generated majorly from the research with the model plant *Arabidopsis thaliana*. Information about molecular features of the CDPK family and its role in important legume crop chickpea is missing. Molecular characterization of the CDPK family will help in understanding their functions in chickpeas.

Here, we have unearthed the entire repertoire of CDPK encoding genes in the chickpea genome. Gene and domain structure analysis confirmed the authenticity and integrity of CDPKs. Phylogenetic analysis and chromosomal localization provided crucial insight into the evolution and expansion of the chickpea CDPK family. Homology modeling was used to understand the three-dimensional structure of chickpea CDPK proteins. Subcellular localization analysis showed that chickpea CDPK proteins are mainly nuclear and/or cytoplasmic. *In-silico* promoter analysis showed the presence of stress, hormone, and development-related *cis*-regulatory elements in CDPK promoters. Extensive expression analysis of the chickpea *CDPK* family was performed under abiotic stresses (drought, salinity, and cold) and during different developmental stages using public RNA-Seq data and qRT-PCR analysis. Expression analysis indicated the involvement of the CDPK family in abiotic stress signaling and plant development in chickpea.

## Materials and Methods

### Identification of CDPKs in the Chickpea Genome

The chickpea genome at National Centre for Biotechnology Information (NCBI) (Varshney et al., 2013) was explored to identify the CDPK encoding genes. CDPK protein sequences of rice and *Arabidopsis thaliana* were retrieved from Uniprot (Swiss-Prot), and used for BLAST homology search in the chickpea genome database. Further, the Hidden Markov Model (HMM) profile of CDPK was extracted from the Pfam (http://pfam.xfam.org/) database and was used as a query to search the chickpea database at NCBI. All obtained sequences were compiled, redundant entries were removed, and only unique entries were used in further analysis. Protein sequences of putative CDPKs were scrutinized for the presence of canonical domains using *in-silico* tools, such as SMART (http://smart.embl-heidelberg.de/), Interpro (https://www.ebi.ac.uk/interpro/), Prosite (https://prosite.expasy.org/) and Pfam (https://pfam.xfam.org/). Various attributes of CDPKs, such as gene ID, protein ID, CDS size, protein size, Introns, molecular weight (MW), isoelectric point (pI), and chromosomal coordinates were extracted from NCBI and ExPASy (https://web.expasy.~org/compute_pi/).

### Phylogenetic Analysis

Multiple Sequence Alignment (MSA) was performed using non-redundant protein sequences of CDPKs from chickpea, *Arabidopsis*, rice, and soybean (*Glycine max*), using Clustal W at default settings. A phylogenetic tree was generated in MEGA X version 10.1.8 (Pennsylvania State University, USA) by the neighbor-joining method. Bootstrap values were calculated in 1,000 replicates to determine the phylogenetic relationship among the CDPKs. The web-server iTOL (Letunic and Bork, 2021) was used to mark the different clades of CIPKs with different colors for better visualization.

### Gene Structures and Domain Prediction

To investigate the gene structure of *CDPKs*, their CDS and the genomic sequences were extracted from NCBI. These sequences were submitted at the Gene Structure Display Server 2.0. (http://gsds.cbi.pku.edu.cn/index.php?input=ite) to generate the gene structure diagram. Identification of domains was carried out using a standalone package of InterPro Scan. Co-ordinates of essential domains and active sites were extracted and used as input in Illustrator for Biological Sequences for the visualization.

### Gene Nomenclature, Chromosomal Localization, and Gene Duplication

The nomenclature of chickpea *CDPK* genes was done based on sequence closeness to their *Arabidopsis* orthologs, and phylogenetic analysis. Genes were named as *CaCDPK* followed by a number (1–22) corresponding to their respective *Arabidopsis* orthologs. The information of chromosome co-ordinates of genes was obtained from NCBI and further used to display chromosomal localization. The MCScanX software package (Wang et al., 2012; Athens, USA) was used to assess the gene duplication within the chickpea *CDPK* gene family. Genes located within 20 kb distance on the same chromosome were considered as tandemly duplicated genes (Feng et al., 2015).

### Homology Modeling of CaCDPK Proteins

The three-dimensional (3D) structures of all CaCDPK proteins were predicted by homology modeling using the PHYRE2 web portal (http://www.sbg.bio.ic.ac.uk/phyre2). PHYRE2 uses advanced remote homology detection methods to build 3D models for protein sequences (Kelley et al., 2015). All the proteins were modeled with 100% confidence by the single highest scoring template model.

### *In-Silico* Subcellular Localization of CaCDPK Proteins

The full-length protein sequences of CaCDPKs were used as input in the subCELlular LOcalization predictor: CELLO online tool (Yu et al., 2006) to predict their subcellular localization.

### Constructs Preparation for *In-Planta* Subcellular Localization

The protein coding sequence (ORF excluding their stop codon) of *CaCDPK5, CaCDPK16*, and *CaCDPK21* genes were amplified from chickpea complementary DNA (cDNA) with gene-specific primers using iProof high fidelity DNA polymerase (Bio-Rad) through PCR in a thermocycler (Applied Biosystems). The list of these primers is given in Supplementary Table 1. The ORFs after amplification cloned into gateway entry vector p-ENTR-D-TOPO (Invitrogen). Genes were subsequently mobilizing into a compatible destination vector pSITE3CA under the control of 2XCaMV35S promoter by LR recombination protocol. The authenticity of all the constructs was ensured by PCR and sequencing.

### Agro-Infiltration Into *Nicotiana benthamiana* and Confocal Microscopy

*Agrobacterium tumefaciens* (GV3101::pMP90) cells were transformed with the YFP constructs of the respective genes. Transformed *Agrobacterium* cells were used to transfect 6-week-old *N. benthamiana* plant leaves. Plants were grown in a growth chamber with the following conditions: 12/12 h photoperiod, 25–26°C temperature, and 60% relative humidity for 48–72 h. Transiently transformed *Nicotiana* leaf discs were analyzed under Total confocal scanner (TCS) SP5 laser scanning electron microscope (Leica, Germany) to detect the florescence, according to Deepika et al. (2022).

### *In-Silico* Promoter Analysis

The 2 kb sequence, upstream of translational start site of *CDPK* genes was retrieved from NCBI. This sequence was used as input in PlantCARE database (http://bioinformatics.psb.ugent.be/webtools/plantcare/html/) for the detection of various *cis*-regulatory elements and motifs. Important *cis-*regulatory related to abiotic stresses, hormonal response, and plant development were selected.

### RNA-Seq Expression Analysis in Developmental Stages and Tissues

To investigate the expression pattern of *CaCDPK* genes during developmental stages, RNA-Seq data was extracted from the NCBI-Sequence Read Archive (SRA) (ID:SRP121085). The raw reads obtained from SRA were processed using the FASTP tool (Shenzhen, China). An index of the reference genome was built and mapping of raw reads onto the reference genome was done using HISAT2 (Kim et al., 2019). StringTie (Pertea et al., 2015) was used to assemble the aligned sequences into potential transcripts. Transcript abundance was calculated by fragments per kilobase of transcript per million reads (FPKM) values. Expression dynamics were analyzed in 27 tissues representing different developmental stages, such as germination (radicle, plumule, and embryo), seedling (Epicotyl and primary root), vegetative (root, petiole, stem, and leaf), reproductive (Petiole, stem, nodules, root, flowers, buds, pods, immature seeds, and leaf), and senescence (immature seeds, mature seeds, seed coat, stem, petiole, root, nodules, leaf, and yellow leaf). Log_2_ transformed expression values were used to generate the heat-map using the MeV4 tool (Maryland, USA). RNA-Seq data for different seed developmental stages (S1–S7) in two distinct desi chickpea varieties (JGK3 and Himchana 1) was extracted from SRA number SRP072563 and SRP072564.

### Plant Growth and Stress Treatment

Desi chickpea (var. ICC4958) was used for gene expression analysis. The seeds were surface sterilized and plants were grown according to Sagar et al. (2020). Ten-day-old seedlings were subjected to different stress treatments. For drought stress, water was withdrawn and seedlings were air-dried within the folds of tissue paper at 22–23°C temperature. Samples were harvested in replicates after 0 (untreated control), 1, 3, and 6 h of drought treatment. For salt stress, seedlings were kept in 150 mM sodium chloride (NaCl) solution in a beaker, and samples were collected after 0, 3, 6, and 12 h. Abscisic Acid (ABA) seedlings were kept in 100 μM (±) ABA in sterile water in a beaker under light and samples were collected after 0, 3, 6, and 12 h treatment. Same aged seedlings were kept in sterile water for control at 22–23°C.

### RNA Extraction and cDNA Synthesis

A total of 100 mg tissue of the control, drought, salinity, and ABA treated root and shoot samples were used for RNA extraction using the TRIzol reagent (Ambion, Life technologies, USA) according to the manufacturer's protocol. The RNA obtained was purified to remove any genomic DNA contamination using an RNeasy Min Elute Clean-up Kit (QIAGEN, Hilden, Germany). The quantity and quality of RNA were ascertained by the ratio 1.8–2.0 for A260:A280 and 2.0–2.3 for A260:A230 using a Nano Drop ONEc (Thermo Scientific, USA) nano-spectrophotometer. Subsequently, MOPS-agarose gel electrophoresis was done to confirm the integrity of the RNA. A total of 1 μg total RNA was used to synthesize the first strand cDNA using a RevertAid first-strand cDNA synthesis kit (Thermo Scientific) according to the manufacturer's protocol.

### Expression Analysis by qRT-PCR

The qRT-PCR primers for selected genes were synthesized by the PRIMER EXPRESS SOFTWARE (Applied Biosystems, USA) according to Singh and Pandey (2015). Their specificity was analyzed using the RGAP BLAST tool and melt curve analysis after a real-time PCR run. The details of all the primers are given in Supplementary Table 1. Three biological replicate samples (with three technical replicates of each biological replicate) of control and nutrient-deficient root and shoots were used to assess the expression pattern. iTaq Universal SYBR Green supermix (Bio-Rad) was used to detect the expression in Bio-rad CFX96 real-time PCR system (Bio-Rad) according to Sagar et al. (2021).

### Statistical Analysis

For statistical significance, all expression and quantitative experiments were replicated three times. The data have been presented as the mean of three replicates ± SD. A two-tailed student's *t*-test was performed to determine the statistical significance among the replicate samples. A *p* < 0.05 was considered statistically significant (denoted by ^*^), *p* < 0.01 (denoted by ^**^) and *p* < 0.005 (denoted by ^***^).

## Results and Discussion

### Identification and Organization of CDPK Family in the Chickpea Genome

**Figure 1:**
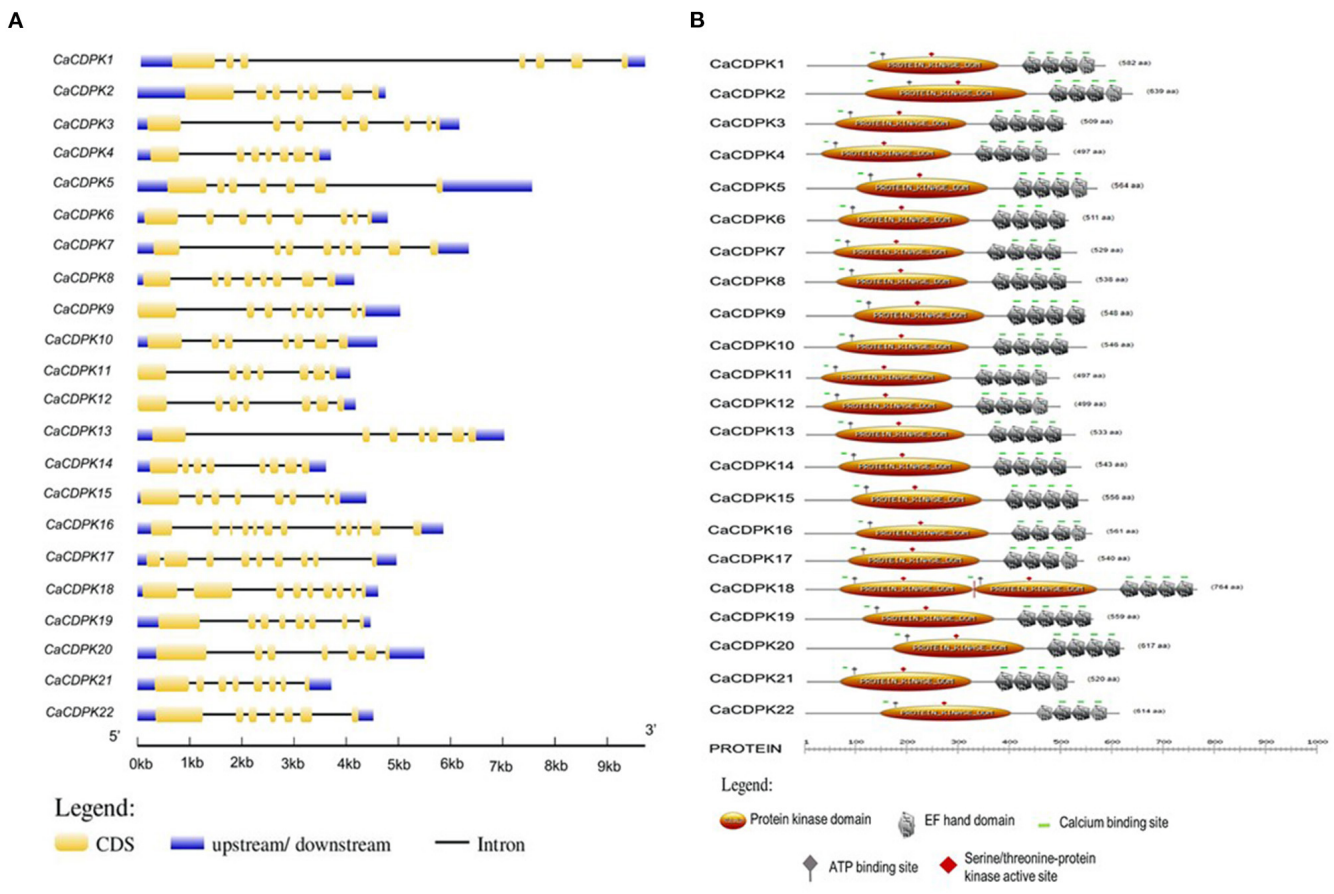
Structural features of the chickpea calcium-dependent protein kinases (CDPK) family. **(A)** Exon-intron organization is shown for *CaCDPK* genes and gene names are mentioned at the left. The scale at the bottom represents gene length in kb. **(B)** Protein domain structure of the chickpea CDPK family is showing conserved protein kinase and EF-hand along with some important sites present. ATP binding site and Serine/threonine-protein kinase active site are located at the protein kinase domain.

A thorough investigation of various databases resulted in the identification of 22 non-redundant CDPK encoding genes in the chickpea genome. This is consistent with previous findings, where different plant species including *Arabidopsis thaliana*, rice (*Oryza sativa*), wheat (*Triticum aestivum L*.), and tomato (*Solanum lycopersicum*) have been found to encode for about 20–30 *CDPK* genes (Cheng et al., 2002; Ray et al., 2007; Li et al., 2008; Hu et al., 2016; Wang et al., 2016). Gene structure analysis showed that *CaCDPKs* are made up of multiple exons and introns. Most *CDPK* genes are comprised of 6-8 introns except *CaCDPK16*, which is comprised of 11 introns. Domain analysis revealed that all the CaCDPKs harbored a canonical catalytic kinase domain toward the N-terminus with a typical Ser/Thr kinase active site and ATP binding site (Figure 1). In addition, four EF-hand motifs were present in all the CDPK proteins toward the C-terminus, which are crucial for Ca^2+^ binding. Strikingly, a crucial Ca^2+^ binding site was found to be missing in one of the EF hand motifs of CaCDPK6, CaCDPK7, CaCDPK8, CaCDPK13, and CaCDPK22. The absence of such an important site might impair the Ca^2+^ binding ability of these CDPKs. However, variations in the number of EF-hand motifs and Ca^2+^ binding sites have also been previously reported in other plants (Cheng et al., 2002; Asano et al., 2005; Kong et al., 2013; Zuo et al., 2013). The in-depth analysis of N-terminal sequences of CaCDPKs revealed that out of 22 proteins, 11 have a myristoylation site (Table 1). The N-terminal domain of many CDPKs in several plant species has been found to contain potential N-myristoylation and N-palmitoylation sites. For example, AtCPK2, AtCPK3, AtCPK6, AtCPK9, AtCPK13, AtCPK5, and AtCPK16 (Benetka et al., 2008; Mehlmer et al., 2010; Lu and Hrabak, 2013), *N. tabacum* NtCDPK2/NtCDPK3 (Witte et al., 2010) and potato (*Solanum tuberosum*) StCDPK4/StCDPK5 (Asai et al., 2013) have been marked with N-myristoylation. These N-terminal modifications determine the membrane targeting of plant CDPKs, as mutations in N-myristoylation or N-palmitoylation sites have been found to hamper their membrane targeting (Singh et al., 2017). CDPKs show a high degree of structural conservation in terms of gene and protein structure, as similar exon-intron and domain arrangement pattern has been observed in diverse plant species including tomato, barley (*Hordeum vulgare* L.), *Brachypodium distachyon*, and *Medicago truncatula* (Hu et al., 2016; Yang et al., 2017; Wen et al., 2020; Zhao et al., 2021). Chickpea CDPK proteins length was found to be in the range of 497–764 amino acids, and molecular weight varied between 56.16 and 68.74 KDa. Interestingly, CDPK protein had a highly variable isoelectric point (pI) falling in the range of 5.09 (CaCDPK22) and 8.99 (CaCDPK16) (Table 1). This indicates that the chickpea CDPK protein might function optimally in a diverse microenvironment.

**Table 1:** Summary of various features of the chickpea CDPK gene family.

**S.No**.	**Gene name**	**Chromosome/Scaffolds**	**Protein ID**	**Exon number**	**CDS size**	**Proten length**	**pI**	**Mol. wt. (KDa)**	**N-Myristoylation**
1	CaCDPK1	Ca4	XP_004498060.1	8	1,749	582	5.21	65.13	Non-Myristoylated
2	CaCDPK2	Ca4	XP_027189268.1	8	1,920	639	5.67	71.12	Non-Myristoylated
3	CaCDPK3	Ca8	XP_004511540.1	8	1,530	509	5.61	57.47	Myristoylated
4	CaCDPK4	Ca4	XP_004496370.1	7	1,494	497	5.33	55.85	N-terminal glycine absent
5	CaCDPK5	Ca2	XP_012568715.1	9	1,695	564	5.8	63.33	Non-Myristoylated
6	CaCDPK6	Ca6	XP_004503656.1	8	1,536	511	5.97	57.53	Non-Myristoylated
7	CaCDPK7	Unplaced scaffold	XP_004513770.1	8	1,590	529	6.62	59.88	Myristoylated
8	CaCDPK8	Ca3	NP_001266123.3	8	1,617	538	6.29	61.11	Myristoylated
9	CaCDPK9	Ca5	XP_004500067.1	8	1,647	548	6.15	61.79	Myristoylated
10	CaCDPK10	Ca7	XP_004508790.1	7	1,641	546	6.1	61.61	Myristoylated
11	CaCDPK11	Ca6	XP_004503860.1	7	1,494	497	5.33	56.16	N-terminal glycine absent
12	CaCDPK12	Ca5	XP_004503020.1	7	1,500	499	5.18	56.58	N-terminal glycine absent
13	CaCDPK13	Unplaced scaffold	XP_004514214.1	7	1,602	533	6.03	59.93	Non-Myristoylated
14	CaCDPK14	Ca6	XP_004507321.1	8	1,632	543	5.88	61.84	Myristoylated
15	CaCDPK15	Ca7	NP_001266075.1	8	1,671	556	5.92	62.15	Myristoylated
16	CaCDPK16	Ca8	XP_004512135.1	12	1,686	561	8.99	63.71	Myristoylated
17	CaCDPK17	Unplaced scaffold	XP_012574838.1	9	1,623	540	5.8	63.33	Myristoylated
18	CaCDPK18	Ca4	XP_004499307.1	9	2,295	764	6.89	86.37	Non-Myristoylated
19	CaCDPK19	Ca2	XP_004491400.1	8	1,680	559	6.46	63.38	Myristoylated
20	CaCDPK20	Ca4	XP_004497622.1	7	1,854	617	5.15	67.77	Non-Myristoylated
21	CaCDPK21	Ca2	XP_004491231.1	8	1,563	520	5.34	58.11	Myristoylated
22	CaCDPK22	Ca2	XP_004489857.1	7	1,845	614	5.09	68.74	Non-Myristoylated

### Evolutionary Analysis of CDPK Family

**Figure 2:**
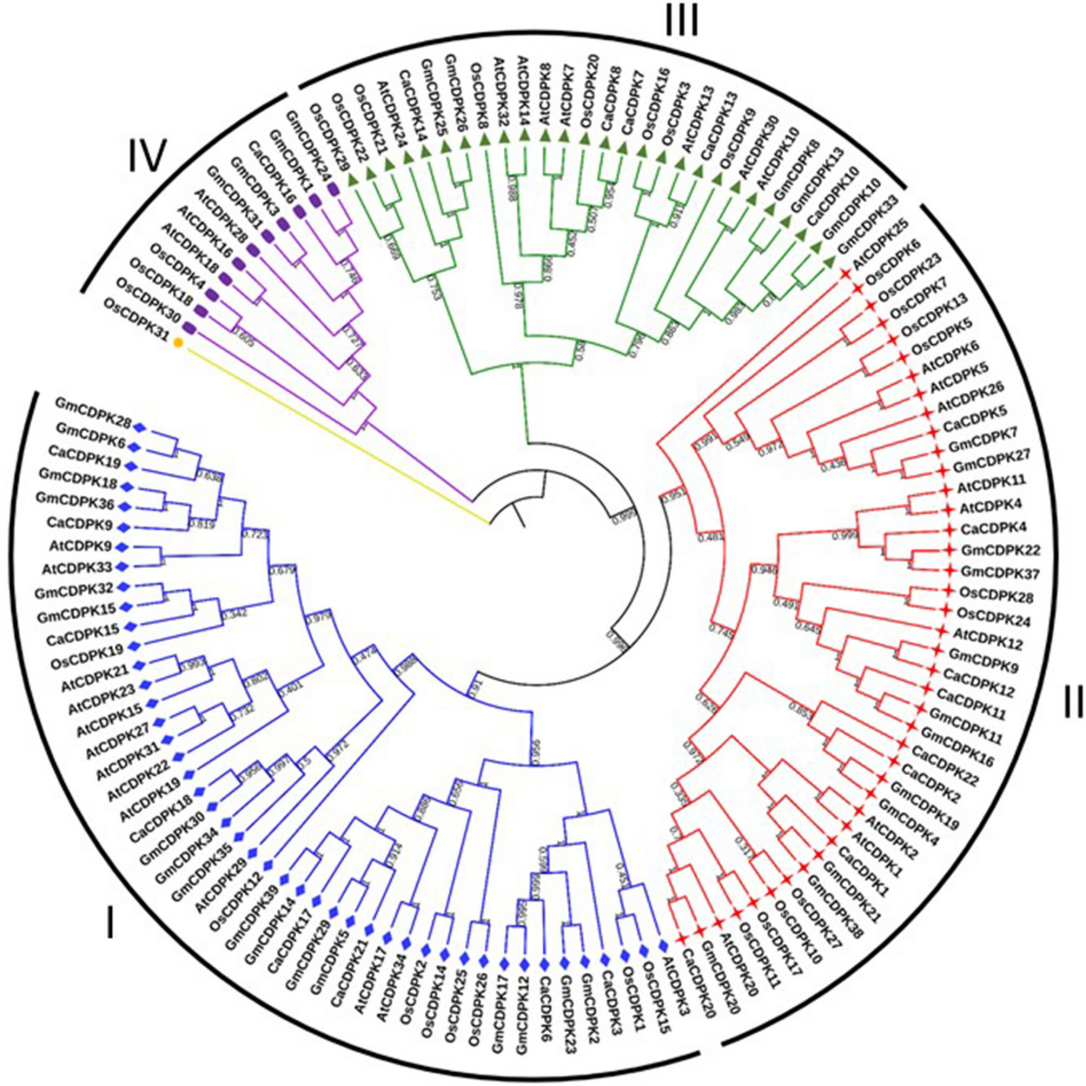
Phylogenetic relationship among CDPKs from different plants. An un-rooted neighbor-joining phylogenetic tree was constructed from the CDPK protein sequences of *Arabidopsis thaliana*, rice, soybean, and chickpea. All the CDPKs could be divided into four groups; I- IV. Numbers above the nodes represent bootstrap values calculated from 1,000 replicates.

To understand the evolution of chickpea CDPKs, phylogenetic analysis was performed with *Arabidopsis*, rice, soybean, and chickpea CDPK proteins. All the CDPKs from different plant species could be demarcated into four sub-clades; group I-IV(Figure 2). While, groups I and II each contained eight chickpea CDPK members, group III contained five CDPKs (CaCDPK7, 8, 10, 13, and 14), and group IV contained only a single CDPK member i.e., CaCDPK16. This phylogenetic distribution is conserved across different plant species, as group I comprises the highest number of CDPK members and group IV comprises the least members (Hu et al., 2016). This analysis suggests the evolutionary conservation of CDPKs across plant species. However, within the separate clades, chickpea CDPKs were closer to the dicot plants *Arabidopsis* and soybean, whereas distantly placed from monocot plant rice. Such distribution indicates the evolutionary divergence of monocot and dicot CDPKs.

**Figure 3:**
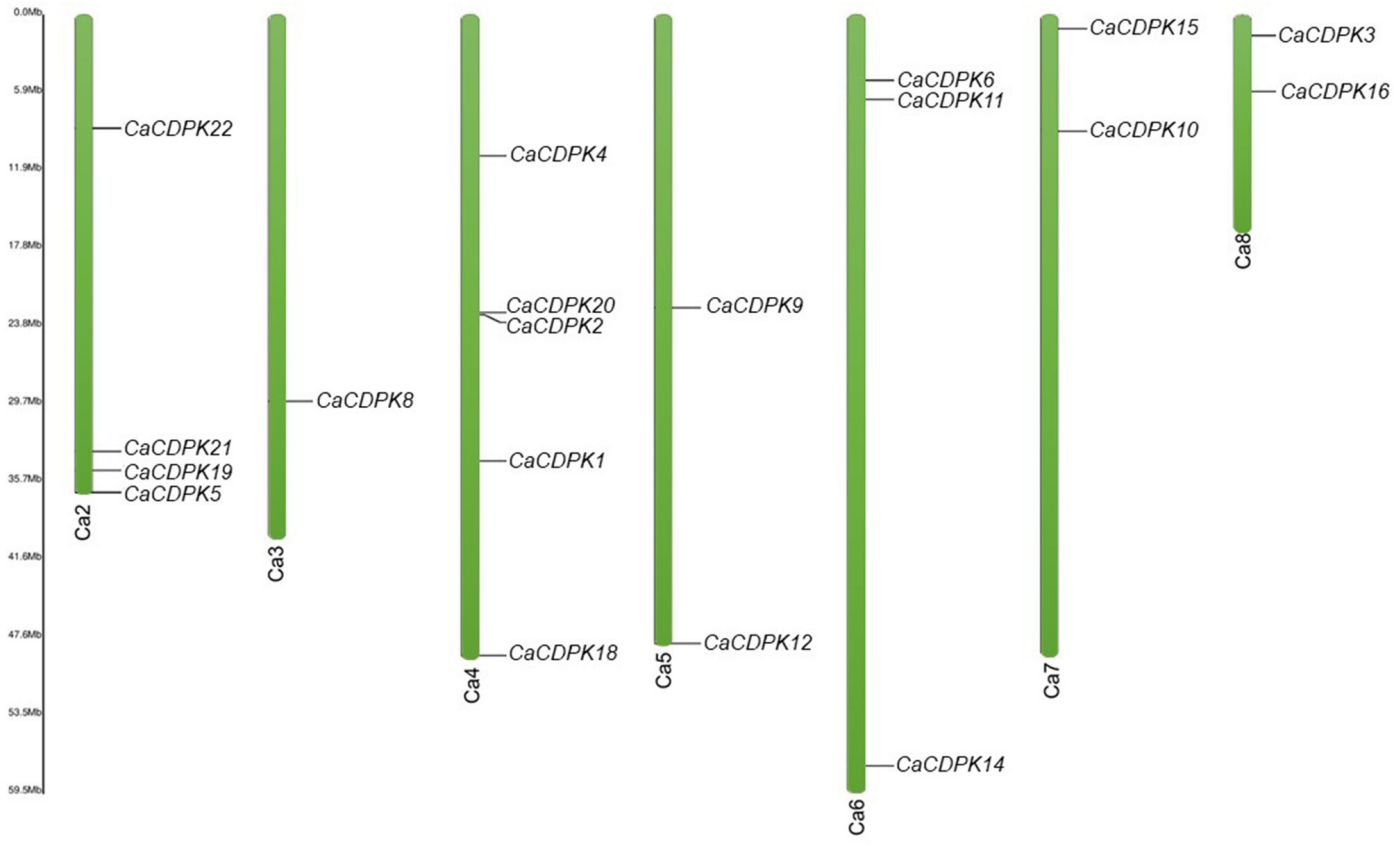
Chromosomal locations of chickpea *CDPK* genes. Green color bars represent the chromosomes, the location of genes has been marked alongside. Chromosome numbers are given at the bottom of each chromosome. Except chromosome 1, the CDPK genes are distributed variably on seven chromosomes.

Chickpea CDPK genes were mapped on the different chromosomes using their chromosomal coordinates. Out of 22 *CDPK* genes, 19 could be mapped on seven out of eight chickpea chromosomes. Three genes (*CaCDPK7, 13*, and *17*) were placed on the scaffold region and none of the genes was localized on chromosome 1. *CaCDPK* genes were variably distributed on chromosomes, with chromosome 4 containing the highest five genes. Four and three *CDPK* genes were located on chromosomes 2 and 6, respectively. Two genes each were located on chromosomes 5, 7, and 8. Whereas, only a single gene, *CaCDPK8* was located on chromosome 3 (Figure 3). Gene duplication analysis revealed that three pairs of *CDPK* genes (*CaCDPK3/6, CaCDPK9/19*, and *CaCDPK11/12*) were segmentally duplicated. In addition, one gene pair, *CDPK2/20* was found to be tandemly duplicated. Gene duplication is considered an important process for the evolution of gene families in plants (Singh et al., 2010, 2014; Sagar et al., 2021). Similar to chickpea, the CDPK family has been evolved through segmental and tandem duplication in diverse plant species, such as *Arabidopsis*, rice, cotton, poplar, moss (*P. patens*), and cabbage (*Brassica rapa*) (Hrabak et al., 2003; Ray et al., 2007; Zuo et al., 2013; Hamel et al., 2014; Liu et al., 2014; Wu et al., 2017). However, the extent of duplication has been found to be varied in different species. For instance, chickpea had only three segmentally duplicated pairs, whereas nine and eight gene pairs were segmentally duplicated in the rice and *Arabidopsis* genomes, respectively (Ray et al., 2007). This indicates that gene duplication is a conserved mechanism of the evolution and expansion of the CDPK family in plants.

### 3D Protein Structure Analysis

**Figure 4:**
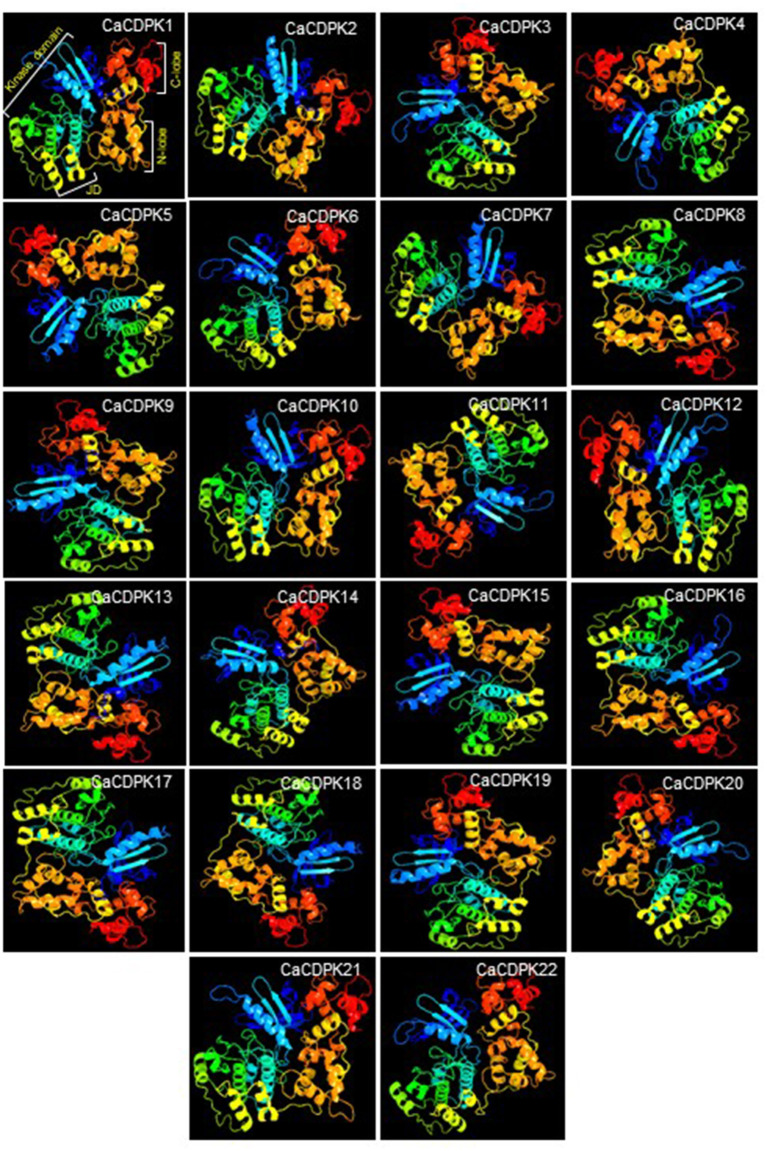
Three-dimensional structure of chickpea CDPK proteins. A three-dimensional (3-D) structure was generated for all 22 members of the chickpea CDPK family. Each CDPK protein is made up of a variable number of α-helix, β-strands, transmembrane helix, and disordered region. Structure comprising blue and green α-helices and β-strands represents kinase domain, yellow helical ribbons represent inhibitory junction domain (JD), orange helices indicate N-lobe, and red helices indicate C-lobe with each containing two elongation factors (EF) hand motifs.

To get an insight into the 3-D protein structure of chickpea CDPKs, homology-modeling was performed for all the CaCDPKs. The protein structures were obtained by comparing with related template protein pbanka-031420 (PDB id 3Q5I). All protein structures were modeled with the template protein with 100% confidence. Most proteins were modeled with a high coverage level ranging from 72 to 90%. The 3-D structures of different CaCDPKs were comprised of a variable number of α-helix, β-strands, disordered region, and transmembrane (TM) - helix (Supplementary Table 2). Among these, the most frequently occurring secondary structure was the α-helix, which warrants the stability of protein structure (Neelamathi et al., 2009). The percentage of α- helices varied from 36 to 48, whereas the β-strands contributed to 11–19% of the protein structure (Supplementary Table 2). In all the CaCDPK proteins, structural folds made up of blue and green α- helices and β-strands represent the catalytic kinase domain. Orange and red color ribbons represent the N-lobe and C-lobe, respectively with each lobe containing two EF hand motifs. Whereas, an inhibitory junction domain (JD) is represented by yellow helical ribbons (Figure 4). Thus, the presence of important characteristic domains and motifs in all the CaCDPK proteins confirms their authenticity and integrity. Similar features of the 3-D structure of CDPK proteins were obtained in different plant species including *Arabidopsis*, rice, maize, ginger (*Zingiber officinale*), and sorghum (Mittal et al., 2017; Vivek et al., 2017). Suggesting that the CDPK protein structure is highly conserved, with similar structural folds and conformational arrangements in higher plants. This also hints toward their conserved functional and structural mechanism.

### Subcellular Localization of CaCDPK Proteins

**Figure 5:**
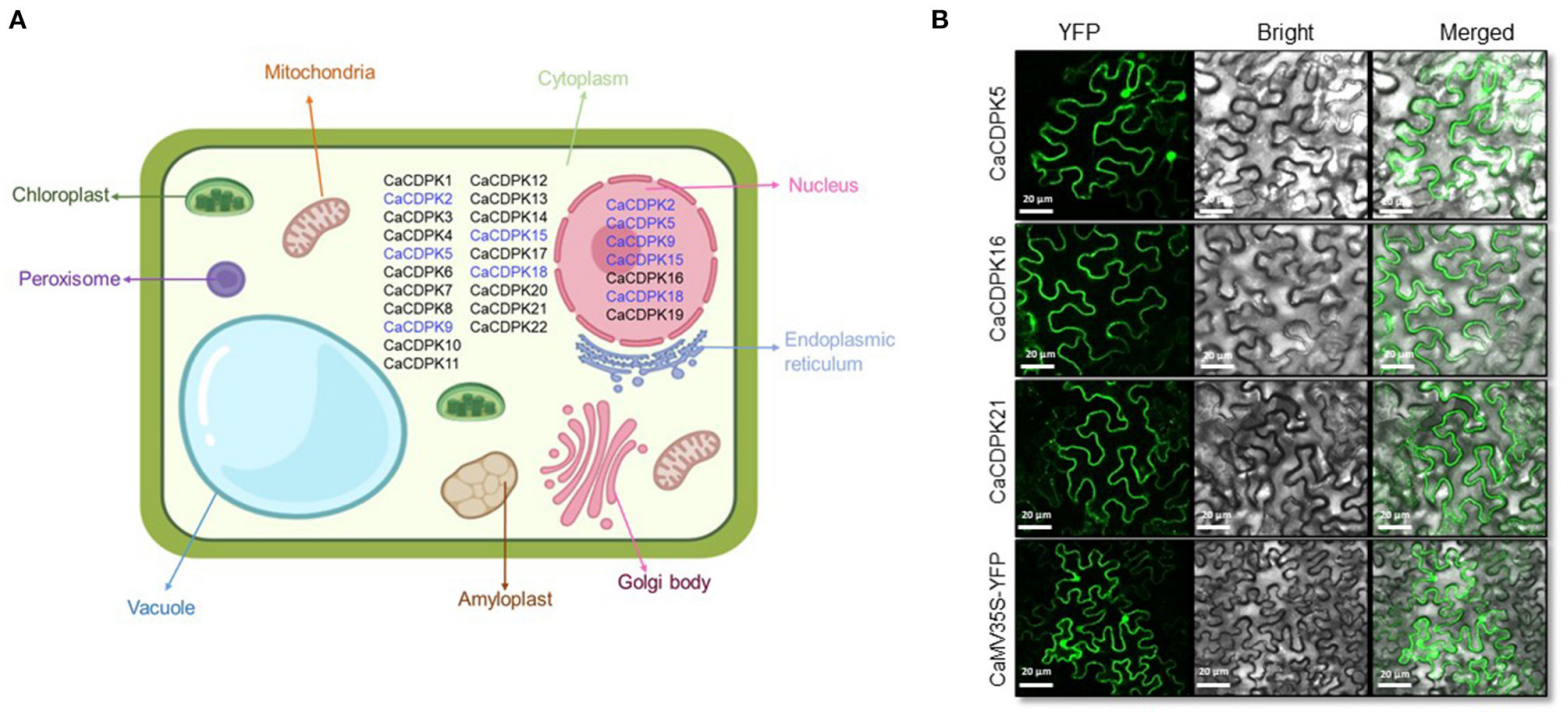
Subcellular localization of chickpea CDPK proteins. **(A)** Localization was predicted using an online tool, CELLO. The figure shows chickpea CDPK proteins localized majorly in the cytoplasm and nucleus based on the highest reliability score with black color. Proteins that were predicted to be localized both in the cytoplasm and nucleus are shown by blue color at both locations. **(B)** *N. benthamiana* cells expressing the YFP- fusion protein driven by the 2XCaMV 35S promoter. Confocal images of fluorescence expressing CaCDPK proteins are showing their variable distribution in different compartments. CaCDPK5 is localized to the cytoplasm and nucleus, whereas CaCDPK16 and CaCDPK21 are localized at the cytoplasm. Cells transformed with vector only (CaMV35S-YFP) are shown in the lowermost row. Scale bar = 20 μm.

To identify the possible sites of activity of CaCDPK proteins in order to have an insight into their function, *in-silico* subcellular localization was performed. By considering the highest reliability score, 15 CaCDPK proteins were exclusively localized in the cytoplasm, whereas only two proteins, CaCDPK16 and 19 were exclusively localized in the nucleus (Figure 5A). Five CDPK proteins, CaCDPK2, 5, 9, 15, and 18 could be localized both in the cytoplasm and nucleus. To validate the *in-silico* localization pattern, fluorescence-based subcellular localization was performed in *N. benthamiana* for three randomly selected candidates; CaCDPK5, 16, and 21. Confocal microscopy analysis revealed that CaCDPK5 was localized in both the nucleus and cytoplasm and CaCDPK21 was localized at the cytoplasm (Figure 5B) which is consistent with *in-silico* prediction. Whereas, CaCDPK16, which was predicted to be nucleus localized was actually localized in the cytoplasm of *Nicotiana* cells. Thus, *in-planta* localization significantly supported and validated the *in-silico* localization pattern. These findings indicate that CaCDPKs might phosphorylate different substrates in the cytoplasm and in the nucleus to regulate various cellular functions in chickpea. CDPKs have been found to be localized at diverse subcellular locations in plants. Most of the CDPKs in Arabidopsis are either membrane-localized or are localized at both cytoplasm and membrane. Whereas, a few AtCDPKs are exclusively cytoplasm localized (Simeunovic et al., 2016). N-myristoylation and palmitoylation are crucial modifications of CDPK proteins that determine CDPK localization (Simeunovic et al., 2016; Zheng et al., 2019). Recently, a study on legume plant *Medicago truncatula* showed that the CDPKs with N-terminal acylation sites were localized at the plasma membrane whereas, those lacking the N-acylation sites were distributed in cytosol and nucleus (Zhao et al., 2021). Consistently, in our study CaCDPK2, 5, and 18 were devoid of N- myristoylation site and distributed in nucleus and cytosol. Surprisingly, several CaCDPK proteins were marked with N- myristoylation site but none of them was found to be localized at the plasma membrane. It is known that N-myristoylation is an irreversible acylation, it requires a second post-translational signal i.e., reversible palmitoylation to sustain membrane localization of a CDPK (Witte et al., 2010). Therefore, the absence of a palmitoylation site in CaCDPK proteins could be a possible reason for them not to be localized at the plasma membrane. Also, the reversibility of the second post-translational signal could lead to the ferrying of CDPK between membrane and cytosol or nucleus (Boudsocq and Sheen, 2013). Moreover, the localization of CaCDPK proteins could be coupled with a stimulus, and a particular condition may lead to a change in their subcellular location. For example, the subcellular location of *Arabidopsis* AtCPK10, AtCPK30, and AtCPK32 proteins was changed due to variable ${\text{NO}}_{3}^{-}$ availability (Liu et al., 2017).

### *cis*-Elements in CDPK Promoters

**Figure 6:**
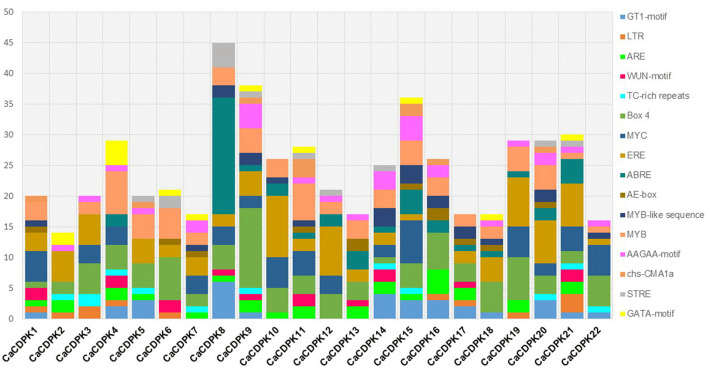
Promoter analysis of chickpea *CDPK* genes. Different *cis*-regulatory elements in the 2 kb upstream promoter region of *CaCDPK* genes are illustrated by different colors in the bar chart. The X-axis represents the name of genes and Y-axis represents the number of different *cis*-elements in each promoter. The names of cis-regulatory elements are mentioned on the right.

*The cis*-regulatory elements in the promoter are crucial for the transcriptional regulation of a gene. Therefore, to get an insight into the transcriptional regulation of *CaCDPK* genes their promoters were investigated for *cis*-regulatory elements. A total of 16 types of *cis*-regulatory elements were found to be variably distributed in *CaCDPK* promoters (Figure 6, Supplementary Table 3). These elements majorly belong to three categories; stress-responsive (GT1-motif, LTR, WUN-motif, ARE, TC-rich repeats, Box-4, AE-box, MYB, Myb-like, MYC, chs-CMA1a, and STRE), hormone-responsive (ABRE, ERE), and plant development related (AAGAA-motif, GATA-motif) (Hughes et al., 2000; Abe et al., 2003; Yamaguchi-Shinozaki and Shinozaki, 2005; Sharma et al., 2011; Nakashima and Yamaguchi-Shinozaki, 2013; Deepika and Singh, 2021; Hou et al., 2021). Abiotic stress-related motif Box-4 (light-responsive) was present in all *CaCDPK* promoters, and MYB motif (drought, low temperature, salt stress-related) was found in all *CaCDPK* promoters except *CaCDPK2*. This suggests that by binding these motifs, specific TFs might regulate the expression of *CaCDPKs* under different abiotic stresses. Markedly, 14 out of 21 *CaCDPK* promoters which contain the MYB motif were also found to contain the ABRE motif (ABA-responsive element), suggesting that these genes could regulate abiotic stresses, such as drought and salinity *via* the ABA-dependent pathway. It has been well-understood that different developmental events and abiotic stresses are interconnected through ABA in plants (Schroeder et al., 2001; Singh et al., 2015). Especially, during the later stages of seed development, a programmed dehydration event is triggered that results in seed dormancy (Hetherington, 2001; Schroeder et al., 2001). ABA commonly regulates these developmental and abiotic stress responses (Nakashima et al., 2009; Hubbard et al., 2010), and genes, which are involved in the regulation of such responses have been found to contain ABRE elements in their promoters (Singh et al., 2012, 2013). Thus, *CaCDPK* promoters with elements like MYB and ABRE could be involved in regulating chickpea plant development under abiotic stress conditions. Apart from two promoters (*CaCDPK4* and *16*), all the *CaCDPK* promoters also contained one or more ethylene-responsive, ERE motifs. Ethylene is an important plant hormone that is involved in plant defense and development (Mishra et al., 2013). The presence of the ERE element in most of the *CaCDPK* promoters reinforces the involvement of the CDPK family in plant development, biotic and abiotic stress responses in chickpea. Consistently, CDPK family members have been found to regulate development during plant-pathogen interaction, herbivore attack, and wounding in diverse plant species (Cai et al., 2015; Zhang et al., 2015; Hettenhausen et al., 2016; Xiao et al., 2017).

### Expression Pattern of *CaCDPKs* in Developmental Stages

**Figure 7:**
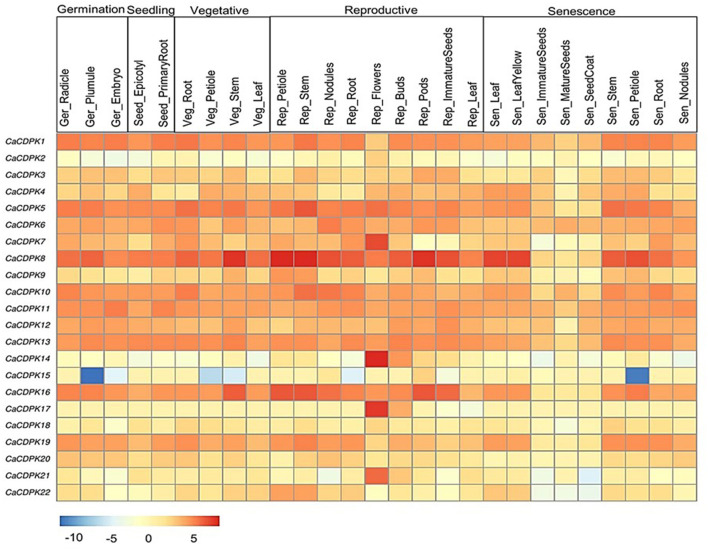
Expression profiles of *CDPK* genes in different developmental stages of chickpea. The heatmap represents the expression pattern of *CaCDPK* genes in different tissues of developmental stages, such as germination, seedling, vegetative, reproduction, and senescence. The genes are named on the left and different tissues/developmental stages are labeled at the top. Scale bar represents the normalized log_2 FPKM values.

To get an insight into the functional role of CDPK genes in plant development, expression analysis was undertaken during various stages of chickpea development. Expression profiles of all *CaCDPK* genes were generated during 27 development stages. These stages represent germination, seedling, vegetative stages, reproductive stages, and senescence. All genes except *CaCDPK2* showed a differential expression pattern during multiple developmental stages (Figure 7, Supplementary Table 4) A total of 10 genes showed ubiquitous expression during most developmental stages these include, *CaCDPK1, 5, 6, 8, 10, 11, 12, 13, 16*, and *19*. Out of these, *CaCDPK8* had the strongest expression during most stages of reproductive development, and some stages of vegetative development and senescence. Notably, the expression of all these 10 *CaCDPK* genes was relatively lower in three stages of senescence namely, immature seeds, mature seeds, and seed coat. This suggests that these *CaCDPK* genes could be involved in a range of developmental processes from germination to senescence. Some of the genes, including *CaCDPK7, 14, 17*, and *21* showed a significant and specific expression in flowers, suggesting their role in flower development. This observation is consistent with previous findings where CDPKs were implicated in flower development. FLOWERING LOCUS T (FT) is an important component of the regulatory network of flower development and it forms a complex with its interdependent partner AtFD (also known as AtbZIP14). *Arabidopsis* CDPK members, AtCPK4/AtCPK6/AtCPK33 phosphorylate AtFD at T282 *in-vitro* in presence of Ca^2+^, which is crucial for the formation and function of this complex. This complex triggers the transcriptional activation of floral meristem identity genes which control floral transition (Abe et al., 2005; Kawamoto et al., 2015). Thus, genes like *CaCDPK7, 14, 17*, and *21* could be involved in similar regulatory networks controlling flower development in chickpea.

In *Arabidopsis*, expression and functional analysis indicated the role of multiple CDPKs in pollen tube growth. Analysis of single and double mutants of *CDPK17* and *CDPK34* revealed that their redundant activity is required for pollen tube tip growth and pollen fitness (Myers et al., 2009). A shaker-type K^+^ inward channel mediates the K^+^ influx which is crucial for pollen tube growth in *Arabidopsis*. CPK11 and CPK24 work together to inhibit this channel, thereby suppressing pollen tube growth (Zhao et al., 2013). Similarly, CPK2 and CPK20 control pollen tube growth *via* regulating another ion channel SLAH3 (Gutermuth et al., 2013). Furthermore, CDPKs regulate plant development via modulating phytohormone signaling. AtCDPK28 regulates the expression of key GA biosynthesis genes (e.g., gene coding for GA3ox1) and thus, controls various facets of plant development, including petiole and stem elongation (Matschi et al., 2013). Similarly, in *Nicotiana attenuata*, NaCDPK4 and 5 regulating GA activity positively regulate stem elongation and plant height (Heinrich et al., 2013). In our study, *CaCDPK15* was specifically and strongly down-regulated during the germination stage of plumule, vegetative stage of petiole and stem, and senescence stage of the petiole. Thus, *CaCDPK15* could be a crucial regulator of the petiole, stem elongation, and plant height in chickpea.

### Expression Pattern in Seed Development in Two Contrasting Chickpea Cultivars

**Figure 8:**
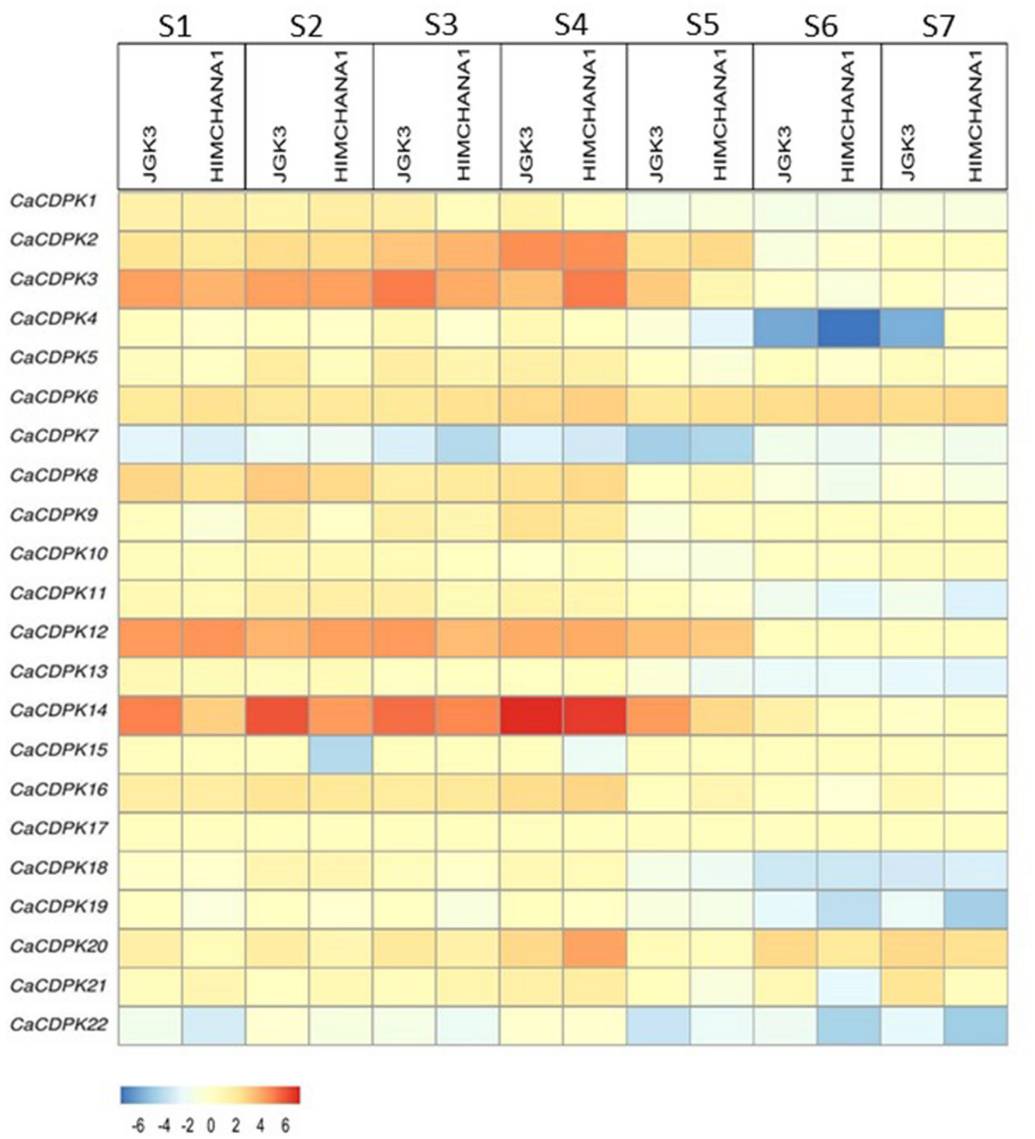
Expression profiles of *CDPK* genes during different stages of seed development in chickpea. Expression profiles are shown for seven seed developmental stages (S1–S7) in two chickpea cultivars: large-seeded JGK3 and small-seeded Himchana1. The genes are named on the left and stages/cultivars are labeled at the top. The scale bar represents the log_2 Fold Change FPKM values.

The seed is the most important part of the chickpea plant from the yield point of view. Therefore, to understand the role of the CDPK family in seed development, expression analysis was performed in two contrasting chickpea cultivars i.e., large-seeded JGK3 and small-seeded Himchana 1. The expression pattern was analyzed during seven stages of chickpea seed development representing early-embryogenesis (S1), mid-embryogenesis (S2), late embryogenesis (S3–S4), mid-maturation (S5), and late-maturation (S6–S7) stages (Rajkumar et al., 2020). Total 17 *CaCDPK* genes expressed differentially in both chickpea cultivars. Out of these, nine genes (*CaCDPK2, 3, 6, 8, 9, 12, 14, 16*, and *20*) were found to be up-regulated, whereas eight genes (*CaCDPK4, 7, 11, 13, 15, 18, 19*, and *22*) were down-regulated in one or more seed stages in both the cultivars (Figure 8, Supplementary Table 5). Notably, most up-regulated *CaCDPK* genes were induced during S1–S4, while their expression was insignificant during later seed stages (S5–S7). In contrast, the most down-regulated genes had significant negative expression during later stages of seed development (S5–S7). This indicates that different sets of *CaCDPK* genes are involved in positively regulating early to late embryogenesis, and negatively regulating mid-late maturation phases of seed development, both in JGK3 and Himchana1 cultivars. *CaCDPK3* and *CaCDPK14* showed similar expression patterns (up-regulation) from the S1–S4 stages with the highest expression in the S4 stage. however, their expression level was higher in JGK3 than Himchana 1. *CaCDPK2* had significant expression during S3 and S4 stages in both the cultivars. *CaCDPK4* showed common as well as cultivar-specific expression as it was specifically down-regulated in the S6 stage of both JGK3 and Himchana1, and S5 and S7 stages of Himchana1 and JGK3, respectively. *CaCDPK15* showed a peculiar expression pattern, it was specifically down-regulated only in S2 and S4 stages of Himchana1. *CaCDPK7* could be a major negative regulator of the entire process of seed development as it was ubiquitously down-regulated during all the seed stages in both chickpea cultivars. Like chickpea, rice orthologs of CDPKs, such as *OsCPK21, OsCPK23*, and *OsCPK31* were found to be strongly up-regulated during different seed developmental stages (Ray et al., 2007). Overexpression of seed-specific *OsCPK31* results in early grain filling, and seed maturation in rice (Manimaran et al., 2015). As suggested by previous reports, CaCDPKs could phosphorylate specific targets/substrates which are important components of the seed development process in chickpea. For instance, a castor (*Ricinus communis*) CDPK protein, RcCDPK2 phosphorylate a sucrose synthase, RcSUS1 at Ser-11 in developing castor oil seed (COS). RcSUS1 cleaves the imported sucrose, thereby, it generated the storage end product in developing COS (Fedosejevs et al., 2016). Similarly, RcCDPK1 *in vivo* phosphorylates Phosphoenolpyruvate carboxylase (PEPC) interacting bacterial-type (BTPC) subunits at Ser-451, and this phosphorylation is inhibitory for BTPC activity. Thus, RcCDPK1 regulates respiratory CO_2_ refixation and anaplerotic photosynthate partitioning in developing COS (Ying et al., 2017).

### qRT-PCR Expression Analysis Under ABA, Drought, and Salinity Stress

**Figure 9:**
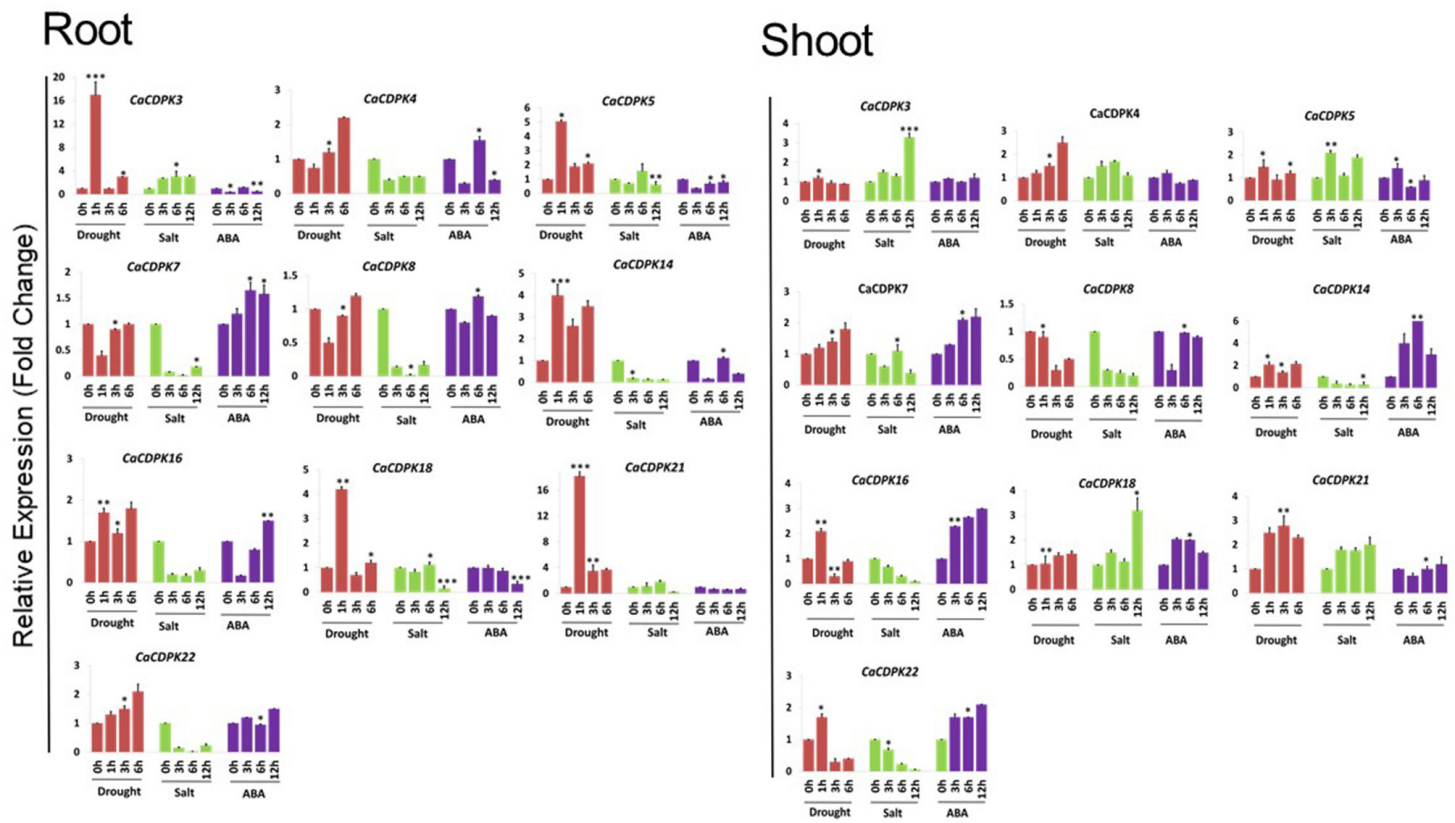
Expression profiles of *CDPK* genes under drought, salt, and ABA treatments in chickpea. qRT-PCR analysis was performed to generate the expression profile of *CaCDPK* genes under drought, salt, and ABA treatment in root and shoot of desi chickpea (ICC4958). Different treatments and time points are indicated on X-axis and the relative expression level of the gene is indicated on Y-axis. Each bar represents the mean value of three replicates. Standard error among the samples is indicated by error bars. *p < 0.05, **p < 0.01 and ***p < 0.005 for treated samples w.r.t. untreated control.

The onslaught of abiotic stresses, such as drought and salinity triggers the increase in cytosolic Ca^2+^ concentration, leading to the generation of stimulus-specific “Ca^2+^ signatures.” These signatures once sensed, trigger specific and overlapping downstream signaling cascades toward an adaptive response. CDPKs are a major class of Ca^2+^ sensors in plant cells that plays a crucial role in the plant's adaptation to stress conditions. Therefore, to understand their role in abiotic stress signaling in chickpea, qRT-PCR analysis was performed for 10 selected genes i.e., *CaCDPK3, 4, 5, 7, 8, 14, 16, 18, 21*, and 22 under abiotic stresses such as drought, salt stress, and phytohormone ABA. Expression profiles were generated at different time points, in root and shoot tissues separately. Based on the expression fold change value ≥1.5 w.r.t. untreated control, all 10 *CaCDPK* genes were found to be differentially expressed under one or more stress conditions (Figure 9, Supplementary Table 6). In roots, except *CaCDPK7* and *8*, eight genes express differentially under drought conditions. *CaCDPK5, 14*, and *21* were significantly up-regulated after 1, 3, and 6 h of drought treatment, while *CaCDPK22* was up-regulated after 3h and 6h of drought treatment. *CaCDPK3* and *16* were exclusively induced after 1h of drought treatment and *CaCDPK4* showed significant up-regulation after 6h drought only (Figure 9). Thus, these *CaCDPK* genes could be categorized as early inducing, late inducing, and ubiquitously expressed. Under ABA treatment, *CaCDPK4, 14, 16, 18*, and *22* were found to be differentially expressed. Drought-induced *CaCDPK4, 16*, and *22* were also up-regulated during the mid (6 h) and late stages (12 h) of the ABA treatment. ABA is known to regulate various aspects of plant growth, including root growth and seed germination (Sagar and Singh, 2019). An overlapping expression of *CaCDPK4, 16*, and *22* under ABA and drought indicates that these CaCDPKs might be involved in regulating root growth *via* the ABA-dependent pathway, and improving water uptake under drought stress. The ABA pathway commonly regulates response to osmotic stresses, including drought and salinity stress (Singh et al., 2016). Numerous studies have revealed the co-regulation of several genes under drought and salinity stress (Ray et al., 2007; Singh et al., 2015, 2017). Surprisingly, in our study, except *CaCDPK3* most drought-induced *CaCDPK* genes were found to be down-regulated under salt stress. Salt stress is known to be inflicted through two components; osmotic and ionic stress (Verslues et al., 2006). The osmotic component is common in both drought and salt stress, whereas the ionic component is unique to salt stress. It is possible that CaCDPKs might regulate salt stress response through its ionic component only, however, it requires detailed functional investigation. Like in roots, *CaCDPK* genes are expressed differentially in one or more conditions in the shoot. Seven genes (*CaCDPK4, 7, 8, 14, 16, 21*, and *22*) showed differential expression under drought stress conditions. *CaCDPK8* was down-regulated in both the stresses and under ABA treatment. Similar to the root, *CaCDPK21* showed a continuous induction (1–6 h) under drought stress, and *CaCDPK4* was induced after 6 h. *CaCDPK22* which showed strong induction after 3 and 6 h drought in roots was found to be up-regulated after 1 h but down-regulated after 3 and 6 h drought treatment. Interestingly, *CaCDPK5* which was continuously expressed in roots showed insignificant expression in shoot whereas, *CaCDPK7* which had insignificant expression in roots was up-regulated after 6 h in the shoot, under drought. This suggests that some CaCDPKs preferentially express in the root or shoot tissues, and possibly regulate tissue-specific functions. Four genes, including *CaCDPK7, 14, 16*, and *22* were found to be up-regulated both under drought stress and ABA treatment. ABA is produced under drought conditions and it regulates the stomata closure. Drought-activated CaCDPKs might phosphorylate and regulate guard cell ion channels to regulate the stomata movement in an ABA-dependent manner, to reduce the transpiration rate and water loss under drought stress in chickpea. Previously, AtCPK3, AtCPK6, AtCPK21, and AtCPK23 have been shown to phosphorylate and regulate SLOW ANION CHANNEL (SLAC1), Ca^2+^ permeable channels, and SLOW ANION CHANNEL-ASSOCIATED (SLAH3) to promote ABA-dependent stomata closure (Mori et al., 2006; Geiger et al., 2010, 2011). Besides phosphorylation, CaCDPKs may also regulate the stomata movement in chickpea through interaction with different proteins, as previously shown by the interaction of AtCPK8 with CATALASE3 (CAT3) and that of OsCPK21 with 14–3–3 protein (OsGF14e) (Zou et al., 2015; Chen et al., 2017). Strikingly, *CaCDPK14* which was strongly induced during most of the seed developmental stages (Figure 8), was also induced under drought and ABA treatment. Careful analysis showed that the promoter of *CaCDPK14* contains an ABRE element. As discussed earlier, seed development and drought stress are interconnected, and ABA is known to simultaneously regulate these processes. Probably by regulating drought response *CaCDPK14* might control seed development *via* the ABA-dependent pathway in chickpea. Such genes could be the potential targets for improving abiotic stress tolerance and crop yield, thus could be of great biotechnological importance.

Unlike roots, at least four genes, including *CaCDPK3, 5, 18*, and *21* were up-regulated under salt stress in the shoot. *CaCDPK21* showed continuous expression after 3, 6, and 12 h of salinity treatment, *CaCDPK5* was induced after 3 and 12 h whereas, *CaCDPK3* and *18* expressed during later stage (12 h) of salt stress (Figure 9, Supplementary Table 6). These CaCDPKs might regulate root-shoot translocation of Na^+^ ion, sequestration of excess Na^+^ from subcellular compartments into the vacuole, and in the maintenance of Na^+^/K^+^ homeostasis in plant cells under salinity stress. CPK3 (Mehlmer et al., 2010) and CPK27 (Zhao et al., 2015) have been shown to regulate Na^+^ toxicity and K^+^ uptake, respectively, in *Arabidopsis* that leads to salt stress tolerance. CDPKs also control oxidative damage or ROS homeostasis to protect the plants from the deleterious effects of stresses, including drought and salinity. For instance, OsCPK12 promotes the expression of ROS scavenger gene *OsAPX2/OsAPX8* and suppresses the expression of NADPH oxidase gene *OsRBOHI*, thereby regulating ROS homeostasis and improving tolerance to salt stress in rice (Asano et al., 2012). Overall, this analysis revealed the spatiotemporal pattern of *CaCDPK* genes expression in chickpea. This hints toward specific and common phosphorylation targets/substrates of CaCDPKs under abiotic stresses in the root and shoot tissues in chickpea.

## Conclusions

In conclusion, a total of 22 non-redundant *CDPK* encoding genes are identified in the chickpea genome. Protein structure and phylogenetic analysis revealed that the CDPK family is highly conserved in terms of structure and evolution, in higher plants. Subcellular localization suggests that most CaCDPK members may target various substrates in the cytoplasm and nucleus to regulate diverse functions. The expression pattern suggests the crucial role of several CDPK members in abiotic stress tolerance and plant development in chickpea. Importantly, some candidate CaCDPKs might regulate drought stress response and seed development. Such genes could be important in determining the crop yield thus, will be of great biotechnological importance. In the future, efforts must be directed for the identification of targets/substrates of key CaCDPKs to delineate CDPK mediated signaling networks underlying abiotic stress response and plant development. Ultimately, knowledge obtained from this study will help in developing abiotic stress-tolerant and high yielding chickpea cultivars that will contribute to world food security.

## Data Availability Statement

The original contributions presented in the study are included in the article/Supplementary Material, further inquiries can be directed to the corresponding author/s.

## Author Contributions

AS and SK conceptualized the study, supervised the project, and designed all the analyses and experiments. DD and NP performed the experiments. DD, NP, SK, and AS analyzed the data. AS, DD, and NP wrote the manuscript. All authors read and approved the final version of manuscript.

## Funding

The authors acknowledge financial support from NIPGR core grant. SK acknowledged the BT/PR40146/BTIS/137/4/2020 project grant from the Department of Biotechnology (DBT), Government of India. AS acknowledges research grant from Science and Engineering Research Board (SERB)—Department of Science and Technology (DST), Government of India (Grant No. EEQ/2018/000106).

## Conflict of Interest

The authors declare that the research was conducted in the absence of any commercial or financial relationships that could be construed as a potential conflict of interest.

## Publisher's Note

All claims expressed in this article are solely those of the authors and do not necessarily represent those of their affiliated organizations, or those of the publisher, the editors and the reviewers. Any product that may be evaluated in this article, or claim that may be made by its manufacturer, is not guaranteed or endorsed by the publisher.
